# Antimicrobial Susceptibility Patterns and Antimicrobial Therapy of Infections Caused by *Elizabethkingia* Species

**DOI:** 10.3390/medicina60121990

**Published:** 2024-12-02

**Authors:** Chienhsiu Huang

**Affiliations:** Department of Internal medicine, Dalin Tzu Chi Hospital, Buddhist Tzu Chi Medical Foundation, Chiayi 62247, Taiwan; hgssport@yahoo.com.tw or dm550671@tzuchi.com.tw; Tel.: +886-9-21552418

**Keywords:** *Elizabethkingia species*, *Meningoseptica*, *Anopheles*, *Miricola*, antimicrobial susceptibility, antimicrobial therapy

## Abstract

*Background and Objectives*: *Elizabethkingia* species have become significant sources of infections acquired in hospital settings and are commonly linked to high mortality rates. Antimicrobial resistance can be influenced by *Elizabethkingia* species, geographical location, antimicrobial susceptibility testing methods, and the time of bacterial isolation. There are distinct antimicrobial susceptibility patterns among species, and the investigation into potential antibiotic susceptibility variations among species is beneficial. There is no guidance on the treatment of *Elizabethkingia* species infections in the literature. Consequently, the purpose of this review was to elaborate on the antimicrobial susceptibility patterns of *Elizabethkingia* species through a scoping review of existing studies on the antibiograms of the *Elizabethkingia* species and on the illness caused by *Elizabethkingia* species. *Materials and Methods*: A comprehensive literature search in PubMed and Web of Science between 1 January 2000 and 30 April 2024 identified all studies, including those that examined antimicrobial susceptibility patterns and antimicrobial therapy of infections caused by *Elizabethkingia* species. I considered studies on antimicrobial susceptibility testing for *Elizabethkingia* species in which only broth microdilution methods and agar dilution methods were used. *Results*: The sensitivity levels of *Elizabethkingia meningoseptica* to piperacillin–tazobactam (5–100%), ciprofloxacin (0–43.4%), levofloxacin (30–81.8%), trimethoprim–sulfamethoxazole (0–100%), tigecycline (15–100%), minocycline (60–100%), and rifampicin (94–100%) varied. The sensitivity levels of *Elizabethkingia anophelis* to piperacillin–tazobactam (3.3–93.3%), ciprofloxacin (1–75%), levofloxacin (12–100%), trimethoprim–sulfamethoxazole (1.02–96.7%), tigecycline (0–52.2%), minocycline (97.5–100%), and rifampicin (20.5–96%) varied. The sensitivity levels of *Elizabethkingia miricola* to piperacillin–tazobactam (41.6–94.0%), ciprofloxacin (14–75%), levofloxacin (77.0–100%), trimethoprim–sulfamethoxazole (18.0–100%), tigecycline (50%), minocycline (100%), and rifampicin (66–85.7%) varied. *Conclusions*: The majority of the isolates of *Elizabethkingia* species were susceptible to minocycline and rifampin. This issue requires professional knowledge integration and treatment recommendations.

## 1. Introduction

*Elizabethkingia* is a genus of gram-negative bacteria. On the basis of 16S rRNA phylogenetic investigations, in 2005, Kim KK et al. classified *Elizabethkingia* within the *Flavobacteriaceae* family [1]. Eight species—*E. meningoseptica*, *E. miricola*, *E. anophelis*, *E. bruuniana*, *E. ursingii*, *E. occulta*, *E. argenteiflava*, and *E. umeracha*—now make up the genus *Elizabethkingia* [2]. The prevalence of infections caused by *Elizabethkingia* species has increased recently in many different countries [3,4,5,6].

Infection caused by *Elizabethkingia* species requires the careful selection of appropriate antimicrobial agents because of the intricate nature of these bacteria. *Elizabethkingia* bacteria have inherent resistance to a wide range of β-lactam antibiotics, β-lactam/lactamase inhibitors, and carbapenems. Two different class B metallo-β-lactamases (MBLs), termed blaBlaB and blaGOB, together with a class A extended-spectrum β-lactamase (ESBL) called blaCME, are responsible for this resistance [7,8,9]. Furthermore, mutations in the topoisomerase IV and/or DNA gyrase genes confer resistance to fluroquinolones [10]. *Elizabethkingia* species do not currently have defined minimum inhibitory concentration (MIC) breakpoints. Instead, susceptibilities are primarily reported via the Clinical and Laboratory Standards Institute M100 guidelines for Enterobacteriaceae and/or the Pharmacokinetic–Pacodynamic “nonspecies” breakpoints from the European Committee on Antimicrobial Susceptibility Testing [11,12].

Ma S et al.’s systematic evaluation revealed that the risk factor for mortality in patients with *E. meningoseptica* infection most frequently mentioned in the literature is inappropriate antibiotic treatment [6]. The only predictor linked to the 14-day survival rate in patients with *E. meningoseptica* bacteraemia was appropriate definite antibiotic use (OR = 0.11, *p* = 0.007), according to the study of Huang YC et al. [13]. Lin YT et al. suggested that the primary risk factor for death in patients with *E. meningoseptica* bacteraemia was the use of inappropriate antibiotics (OR = 3.76, *p* = 0.028) [14]. In a multivariate analysis, Lin JN et al. reported that inappropriate empirical antimicrobial therapy was a risk factor for mortality in patients with infections caused by *Elizabethkingia* species (OR = 12.45, *p* = 0.027) [15]. The use of appropriate antimicrobial therapy is critical for the effective management of infections caused by *Elizabethkingia* species. However, antimicrobial resistance can be influenced by *Elizabethkingia* species, geographical location, antimicrobial susceptibility testing methods, and the time of bacterial isolation. *Elizabethkingia* species exhibits variable susceptibility to fluoroquinolones, trimethoprim–sulfamethoxazole, piperacillin/tazobactam, rifampicin, minocycline, and tigecycline [16,17]. Accurate identification of *Elizabethkingia* species to species level is critical in therapeutic settings. There are distinct antimicrobial susceptibility patterns among species, and investigation into potential antibiotic susceptibility variations among species is beneficial. There is no guidance on the treatment of *Elizabethkingia* species infections in the literature. Consequently, the purpose of this review was to elaborate on the antimicrobial susceptibility patterns of *Elizabethkingia* species through a scoping review of existing studies on the antibiograms of the *Elizabethkingia* species and on the illness caused by *Elizabethkingia* species.

## 2. Materials and Methods

A comprehensive literature search in PubMed and Web of Science between 1 January 2000 and 30 April 2024 identified all studies, including those that examined antimicrobial susceptibility patterns of *Elizabethkingia* species. The phrases “*Elizabethkingia* or *Elizabethkingia meningoseptica* or *Elizabethkingia anophelis* or *Elizabethkingia miricola*” in connection with “antimicrobial susceptibility” or “antimicrobial therapy”. I checked and evaluated each study for eligibility. After eliminating duplicates, I analyzed the titles and abstracts of all the retrieved papers to identify qualifying records. After eliminating irrelevant studies, the eligibility of all relevant papers was determined by reading their full texts. Information on the author, year of publication, country, and total number of antimicrobial susceptibility patterns was extracted from the full text articles. In the current scoping review, I considered randomized controlled trials, prospective studies, and retrospective studies. Only studies that explicitly examined the antimicrobial susceptibility patterns of *Elizabethkingia* species were included. Only English articles were included.

Owing to their differences, *Elizabethkingia* species may have different antimicrobial susceptibilities, and little information exists regarding the antimicrobial susceptibility of *Elizabethkingia* species in the literature. In vitro testing has been performed for numerous antibiotics. Because different *Elizabethkingia* species, testing procedures, and interpretation standards may be used in different studies, antibiotic susceptibilities may not be comparable. The disk diffusion method is an inexpensive, convenient, and excellent antimicrobial susceptibility test approach for many microorganisms. Only ceftazidime, minocycline, levofloxacin, and rifampin were considered acceptable for the antimicrobial susceptibility test executed through the disk diffusion method. In the E-test method, the antibiotic gradient diffusion strips constitute a convenient tool for antimicrobial susceptibility test. The E-test method underestimated the MICs of all antimicrobial agents tested, except for rifampin. The tendency of the E-test to underestimate MICs has been reported for other microorganisms [18,19]. Disparities between automated systems and broth microdilution methods have been documented. Disk diffusion and E-test susceptibilities, particularly for vancomycin and piperacillin–tazobactam, have been shown to be unreliable and imprecise for *Elizabethkingia*, and broth microdilution is advised as a more accurate method of determining susceptibilities. Therefore, the gold standard for determining the MICs for *Elizabethkingia* species is the use of broth microdilution procedures [20,21,22]. The findings of antimicrobial susceptibility testing for *E. anophelis* via three assays (agar dilution, disk diffusion, and E-tests) were examined in a study by Chiu CT et al. The overall categorical agreement between the disk diffusion and agar dilution techniques was 74.8%. The E-test and agar dilution assays had an overall categorical agreement rate of 76.1%. The author concluded that the findings of antimicrobial susceptibility testing for various antibiotics acquired via disk diffusion and E-test techniques varied considerably from those obtained via the agar dilution method. It is not advisable to use these two methods as a regular substitute for antimicrobial susceptibility testing for *E. anopheles* [23]. Therefore, there are discrepancies in the findings of antimicrobial susceptibility tests for *Elizabethkingia* species using different methodologies. In the present review, I considered studies on antimicrobial susceptibility testing for *Elizabethkingia* species in which only broth microdilution methods and agar dilution methods were used.

## 3. Results

**Figure 1:**
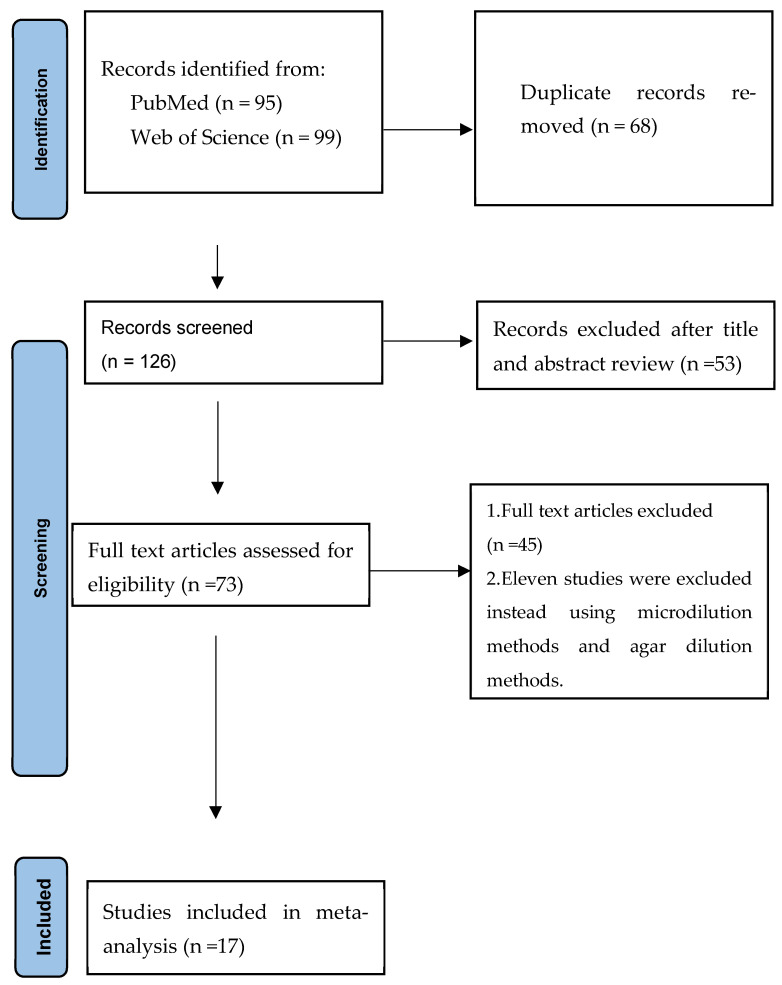
Flow diagram of the study selection process. Legend: seventeen studies were included in this scoping review.

The details of the study selection process are shown in Figure 1. After excluding duplicates and irrelevant studies, 73 potentially relevant articles remained. After full text article review, 45 articles were excluded because they lacked results of the antimicrobial susceptibility patterns of *Elizabethkingia* species. Eleven studies were excluded instead using microdilution method and agar dilution method [3,6,14,16,24,25,26,27,28,29,30]. Ultimately, 17 studies were included in the present scoping review [5,15,17,20,21,22,31,32,33,34,35,36,37,38,39,40,41]. All of the included studies were retrospective studies and had a high risk of bias.

Four investigations were conducted to explore the antimicrobial susceptibility of *Elizabethkingia* species [15,31,32,33]. The sensitivity levels of *Elizabethkingia* species to piperacillin/tazobactam (8.5–86.5%), ciprofloxacin (9.8–50%), levofloxacin (52.2–80.8%), trimethoprim-sulfamethoxazole (12.0–90.0%), tigecycline (23.9–78.8%), minocycline (91.3–100%), and rifampicin (76.9%) varied (Table 1).

Seven investigations were conducted to explore the antimicrobial susceptibility of *E. meningoseptica* [5,15,17,20,22,32,34]. The sensitivity levels of *E. meningoseptica* to piperacillin-tazobactam (5–100%), ciprofloxacin (0–43.4%), levofloxacin (30–81.8%), trimethoprim-sulfamethoxazole (0–100%), tigecycline (15–100%), minocycline (60–100%), and rifampicin (94–100%) varied (Table 2).

Twelve investigations were conducted to explore the antimicrobial susceptibility of *E. anophelis* [15,17,20,21,22,32,34,35,36,37,38,39]. The sensitivity levels of *E. anophelis* to piperacillin-tazobactam (3.3–93.3%), ciprofloxacin (1–75%), levofloxacin (12–100%), trimethoprim-sulfamethoxazole (4–96.7%), tigecycline (0–52.2%), minocycline (97.5–100%), and rifampicin (20.5–96%) varied greatly (Table 3).

Five investigations were conducted to explore the antimicrobial susceptibility of *E. miricola* [17,22,32,34,40]. The sensitivity levels of *E. miricola* to piperacillin–tazobactam (41.6–94.0%), ciprofloxacin (14–75%), levofloxacin (77.0–100%), trimethoprim–sulfamethoxazole (18–100%), tigecycline (50%), minocycline (100%), and rifampicin (66–85.7%) varied (Table 4).

## 4. Discussion

### 4.1. Antimicrobial Susceptibility Patterns of Elizabethkingia Species

The results of the comprehensive analysis of the antimicrobial susceptibility of *Elizabethkingia* species varied by geographical location. The susceptibility to tigecycline was rather low, and no studies have shown that antimicrobial susceptibility is greater than 80% [15,31,33]. The susceptibility to ciprofloxacin and levofloxacin was rather low, except in the study of Comba IY et al. (2020, USA, levofloxacin 80.8%) [32]. The susceptibility to levofloxacin was greater than that to ciprofloxacin. Levofloxacin with the C-8 methoxy group has greater antibacterial action against fluoroquinolone-resistant bacteria that carried the gyrA mutation, which might account for some of the observed disparity [42,43]. The susceptibility to trimethoprim–sulfamethoxazole was rather low, except in the study of Comba IY et al. (2020, USA, 90.0%) [32]. The susceptibility to piperacillin–tazobactam varied. Two studies reported that the susceptibility was greater than 80% [31,32], but two other studies reported that the susceptibility was rather low [15,33]. The study by Wang C et al. (2020, China) reported that the sensitivity levels of *Elizabethkingia* species to rifampicin was 76.9% [31]. According to the present review, the effects of levofloxacin, trimethoprim–sulfamethoxazole, piperacillin–tazobactam and rifampicin therapy in patients with infections caused by *Elizabethkingia* species are questionable. Treating patients with infections caused by *Elizabethkingia* species with tigecycline, and ciprofloxacin is not recommended.

Most *Elizabethkingia* species responded well to minocycline, and all of the studies reported that antimicrobial susceptibility was greater than 90%. Lin JN et al. (2018, Taiwan) and Wu C et al. (2024, China) reported that only minocycline was effective against *Elizabethkingia* species [15,33]. Wang L et al. (2020, China) and Comba IY et al. (2022, USA) reported that minocycline was effective against *Elizabethkingia* species [31,32]. Minocycline tends to be the preferred treatment for patients with infections caused by *Elizabethkingia* species.

### 4.2. Antimicrobial Susceptibility Patterns of E. meningoseptica

The results of the comprehensive analysis of the antimicrobial susceptibility of *E. meningoseptica* varied by geographical location. The use of vancomycin, which is clinically ineffective for treating *Elizabethkingia* species infections, as a monotherapy is consistent with increased MICs of ≥4 mg/mL. Many laboratories do not test for vancomycin susceptibility in *Elizabethkingia* species because of these problems and the absence of evidence about microbiological testing that supports tests from breakpoint-setting organizations [44,45]. Vancomycin is not advised for use because of its high MIC, despite some anecdotal reports showing that it can successfully cure meningitis caused by *E. meningoseptica* when combined with other antibiotics [44,45,46,47]. The susceptibility to ciprofloxacin was rather low [5,15,17,20,32]. Ciprofloxacin was not effective against *E. meningoseptica*. A study by Hsu MS et al. (2011, Taiwan) revealed that susceptibility to levofloxacin was greater than 80% (81.8%) [5]. The susceptibility to levofloxacin was 71.4% and 78.7% in the studies of Kuo SC et al. (2021, Taiwan) and Comba IY et al. (2022, USA), respectively [20,32]. Three other studies reported that the susceptibility to levofloxacin was greater than 60% [15,17,22]. Patients with *E. meningoseptica* bacteraemia treated with fluoroquinolone had a lower mortality rate than did those treated with nonfluoroquinolone (tazobactam/piperacillin, trimethoprim/sulfamethoxazole, and minocycline), according to a retrospective study conducted in Taiwan in 2018 [48]. A Taiwanese study revealed 93 cases of *E. meningoseptica* bacteraemia and 51 cases of levofloxacin-resistant bacteraemia. The 14-day mortality rate for patients with the levofloxacin-resistant strain of *E. meningoseptica* bacteraemia was significantly greater than that for those with the levofloxacin-susceptible strain [13]. However, a different Taiwanese study (2018) revealed that *E. meningoseptica* has a significant frequency of fluoroquinolone-targeting gene mutations [15]. The present review suggests that fluoroquinolones are not recommended for treating *E. meningoseptica* infection. The susceptibility to tigecycline was rather low, except in the study of Kuo SC et al. (100%, 2021, Taiwan) [20]. The investigation conducted by Kuo SC et al. included only seven isolates of *E. meningoseptica*. Treating patients with infections caused by *E. meningoseptica* with tigecycline is not recommended. The susceptibility to piperacillin–tazobactam was 100% and 89.4% in the studies of Han MS et al. (2017, Korea) and Comba IY et al. (2022, USA), respectively [17,32]. Four studies from Taiwan reported that the susceptibility to piperacillin-tazobactam was less than 80% [5,15,20,22]. Piperacillin–tazobactam may be suitable for treating *E. meningoseptica*, but piperacillin–tazobactam is not recommended for treating *E. meningoseptica* infection in Taiwan. The susceptibility to trimethoprim–sulfamethoxazole varies greatly. Trimethoprim–sulfamethoxazole was proposed by Hsu MS et al. (2011, Taiwan) as a possible therapy for *E. meningoseptica*-related infection [5]. Trimethoprim–sulfamethoxazole was proposed as a potential guiding treatment for *E. meningoseptica* infections by Comba IY et al. (2022, USA) [32]. Three other studies reported the susceptibility to trimethoprim–sulfamethoxazole was less than 20% [15,17,22]. According to the present review, the effect of trimethoprim-sulfamethoxazole treatment in patients with infections caused by *E. meningoseptica* is questionable.

Three studies reported that the majority of isolates responded well to minocycline (100%) [20,22,32]. The study of Lin JN et al. revealed that the antimicrobial susceptibility was only 60%. The author recommended more stringent empirical antibiotic treatment for infections caused by *E. meningoseptica* [15]. A study by Cheng YH revealed that the susceptibility of *E. meningoseptica* to minocycline was 100%. The author suggested that minocycline was effective against *E. meningoseptica* [22]. Patients with *E. meningoseptica* infection may benefit the most from minocycline therapy. Chang TY et al. reported that rifampicin was effective against *E. meningoseptica*, which was 100% susceptible. Rifampicin was the most active agent in Chang TY’s study [34]. Rifampicin effectively suppressed *E. meningoseptic* infection in vitro. However, medical experts need to explore why *E. meningoseptica* responds well to rifampicin.

### 4.3. Antimicrobial Susceptibility Patterns of E. anophelis

The results of the comprehensive analysis of the antimicrobial susceptibility of *E. anophelis* varied by geographical location. The susceptibility to tigecycline was rather low [15,20,21,22,35,37,38,39]. Tigecycline is not recommended for treating *E. anophelis* infection. The susceptibility to ciprofloxacin was rather low [15,17,20,21,22,32,35,36,37,38,39]. The susceptibility to levofloxacin varied [15,17,20,21,22,32,35,36,37,38,39]. The only study by Burnard D et al. (2020, Australia) revealed that the susceptibility to levofloxacin was greater than 80% (100%) [36]. The susceptibility to levofloxacin was 78.5%, 70.0%, 76.1%, and 71.4% in the studies of Chew KL et al. (2018, Singapore), Tang HJ et al. (2021, Taiwan), HU S et al. (2022, USA), and Comba IY et al. (2022, USA), respectively [32,35,38,39]. Six other studies reported that the susceptibility to levofloxacin was less than 60% [15,17,20,21,22,37]. Perin A (2017, USA) employed the disk diffusion technique to determine antimicrobial susceptibility and reported that the susceptibility to fluoroquinolone was greater than 80% (ciprofloxacin 92% and levofloxacin 96% [3]. These antimicrobial susceptibility testing results obtained via the disk diffusion method are practically contrary to those of the present review. The present review suggests that fluoroquinolones, especially ciprofloxacin, are not recommended for treating *E. anophelis*.

The susceptibility to piperacillin–tazobactam was 92%, 92.4%, 88%, 93.3%, and 92.9% in the studies of Han MS et al. (2017, Korea), Chew KL et al. (2018, Singapore), Jian MJ et al. (2019, Taiwan), Tang HJ et al. (2021, Taiwan), and Comba IY et al. (2022, USA), respectively [17,21,32,35,38]. The susceptibility to piperacillin/tazobactam was 73% and 71.8% in the studies of Cheng YH et al. (2019, Taiwan) and Chang Y et al. (2021, China), respectively [22,37]. Four other studies reported that the susceptibility to piperacillin-tazobactam was less than 70% [15,20,36,39]. Piperacillin-tazobactam may be suitable for treating *E. anophelis* infection. The present review revealed that the susceptibility to trimethoprim–sulfamethoxazole varies greatly [15,17,20,21,22,32,35,36,38,39]. The susceptibility to trimethoprim–sulfamethoxazole was 96.7%, 85.7%, and 92.4% in the studies of Kuo SC et al. (2021, Taiwan), Comba IY et al. (2022, USA), and Chew KL et al. (2018, Singapore), respectively [20,32,35]. The susceptibility to trimethoprim–sulfamethoxazole was 75% and 73.3% in the studies of Burnard D et al. (2020, Australia) and Tang HJ et al. (2021, Taiwan), respectively [36,38]. Five other studies reported that the susceptibility to trimethoprim–sulfamethoxazole was less than 70% [15,17,21,22,39]. The only study that proposed trimethoprim–sulfamethoxazole as a guiding treatment for *E. anophelis* infection was that of Comba IY et al. (2022, USA) [32]. According to the present review, treating patients with *E. anophelis* infection with trimethoprim–sulfamethoxazole is questionable.

Ten studies showed that the majority of isolates responded well to minocycline [15,20,21,22,32,35,36,37,38,39]. According to Lin JN et al. (2018, Taiwan), minocycline may be the recommended medication for those with *E. anophelis* infection [15]. Chang Y et al. (2021, China) proposed that minocycline be considered a treatment option for infections caused by *E. anopheles* [37]. A study by Cheng YH et al. (2018, Taiwan) revealed that the susceptibility of *E. anophelis* to minocycline was 98%. The author suggested that minocycline was effective against *E. anopheles* [22]. Jian MJ et al. (2019, Taiwan) reported that the antimicrobial susceptibility of *E. anaphelis* to minocycline was 100%. The author suggested that minocycline has potential for the treatment of *E. anaphelis* infection [21]. The isolates of *E. anophelis* in Chang Y et al.’s research from 2021 (China) presented high in vitro sensitivity to minocycline (100%). The author concluded that minocycline is the best antibiotic for *E. anophelis* in vitro [37]. The present review suggests that minocycline has the potential to be the preferred treatment for patients with *E. anophelis* infection.

Six studies reported the susceptibility of *E. anophelis* to rifampicin [17,20,34,37,38,39]. Five studies revealed that nearly all isolates of *E. anophelis* were significantly susceptible to rifampicin, with the exceptions of the study of Chang Y et al. (20.5%, 2021, China) [37]. Rifampicin is effective against *E. anophelis*, according to Kuo SC et al. (2021, Taiwan), with an 81.1% susceptibility [20]. A study by Hu S et al. (2022, USA) revealed that the antimicrobial susceptibility to rifampicin was 93.9% [39]. A study by Chang TY (2019, Taiwan) et al. revealed that the susceptibility of *E. anophelis* to rifampicin was 94.4% [34]. Rifampicin is a promising therapeutic option for treating *E. anophelis* infection.

### 4.4. Antimicrobial Susceptibility Patterns of E. miricola

The antimicrobial susceptibility of the recently reported *E. miricola* is poorly understood. The results of the comprehensive analysis of the antimicrobial susceptibility of *E. miricola* varied by geographical location. The susceptibility to tigecycline and ciprofloxacin was rather low. Ciprofloxacin and tegicycline are not recommended to treat *E. miricola* infection. A study by Chang TY et al. revealed that the susceptibility of *E. miricola* to rifampicin was 85.7%, suggesting that rifampicin could be a promising treatment for *E. miricola* [34]. However, a study by Han MS et al. revealed that the susceptibility of *E. miricola* to rifampicin was 66% [17]. The susceptibility to piperacillin–tazobactam was 94% and 91.7% in the studies of Han MS et al. (2018, Korea), and Comba IY et al. (2022, USA), respectively [17,32]. However, two other studies reported that the susceptibility of *E. miricola* to piperacillin-tazobactam was less than 80% [22,40]. The susceptibility to trimethoprim–sulfamethoxazole was 100% and 83.3% in the studies of Kenna DTD et al. (2018, UK) and Comba IY et al. (2022, USA), respectively [32,40]. However, two other studies reported that the susceptibility of *E. miricola* to trimethoprim–sulfamethoxazole was less than 30% (28% and 18%) [17,22]. According to the present review, the effects of treatment with rifampicin, piperacillin/tazobactam, and trimethoprim–sulfamethoxazole in patients with infections caused by *E. miricola* are questionable. All isolates of *E. miricola* were significantly susceptible to minocycline in a study by Kenna DTD et al. [40], and a study by Cheng YH et al. reported that minocycline was effective against *E. miricola* [22]. Minocycline may be suitable for treating *E. miricola* infection. The susceptibility to levofloxacin was 100% and 91.7% in the studies of Han MS et al. (2018, Korea) and Comba IY et al. (2022, USA), respectively [17,32]. Another study reported that the susceptibility of *E. miricola* to levofloxacin was 77% [22]. Levofloxacin may be suitable for treating *E. miricola* infection.

### 4.5. Antimicrobial Susceptibility Patterns of Other Elizabethkingia Species

The antimicrobial susceptibility of the recently reported *E. bruuniana*, *E. ursingii*, and *E. occulta* strains is still poorly understood. Six *E. bruuniana* strains that were isolated from a clinical specimen in Taiwan were reported in a recent investigation [41]. All of these isolates were responsive to minocycline, and all of these isolates were resistant to trimethoprim/sulfamethoxazole. The majority of these isolates were susceptible to levofloxacin (67%) and tigecycline (67%). The susceptibility to ciprofloxacin (33%) was comparatively low.

## 5. Conclusions

Patients with infections caused by *Elizabethkingia* species require prompt identification and effective antibiotic treatment since these species are typically resistant to multiple antibiotics and have variable susceptibility patterns. The present review provides treatment recommendations for patients with infections caused by *Elizabethkingia* species (Table 5). The majority of the isolates of *Elizabethkingia* species reported in the literature are minocycline susceptible. When treating patients with infections caused by *Elizabethkingia* species, minocycline has the potential to be the preferred treatment. In addition, according to the literature, rifampin may be a promising candidate drug of choice for infections caused by *Elizabethkingia* species.

## 6. Future Directions

The present review recommends that trimethoprim–sulfamethoxazole as a guiding treatment for patients with infections caused by *Elizabethkingia* species is questionable. Minocycline and rifampin were shown to effectively suppress *Elizabethkingia* species in vitro. In the review of Lee LY et al., it was described that cefiderocol may serve as an alternative due to its low minimum inhibitory concentration against *E. anopheles* [49]. In the study of Feng M et al., the susceptibility of *Elizabethkingia* species to ceftazidime–avibactam was 33.3% [50]. There is no consensus on the effective treatment for *Elizabethkingia* species infections due to their relative scarcity, and conducting a clinical trial is difficult. Further research is necessary to determine the most effective antimicrobial agents to treat patients with infections caused by *Elizabethkingia* species.

According to Seong et al.’s study, 187 patients received combination treatment (mortality rate: 25.6%), and 23 patients received monotherapy (mortality rate: 21.7%) for *Elizabethkingia* species infections. There were no significant differences between the groups [51]. According to Chan JC et al.’s study, combination therapy with trimethoprim–sulfamethoxazole, piperacillin/tazobactam, or a fluoroquinolone was successful at a rate of 81.8% in 13 pediatric patients with *E. meningoseptica* infections [29]. In the study of Wei Q et al., a case of combination therapy of trimethoprim–sulfamethoxazole and eravacycline for treating *E. anophelis* pneumonia with a fully recovered state was reported [52]. The therapeutic effectiveness of combination treatment for infections caused by *Elizabethkingia* species has rarely been studied in the literature. This issue requires professional knowledge integration and treatment recommendations.

## Figures and Tables

**Table 1:** Antimicrobial susceptibility patterns of *Elizabethkingia* species.

Author/Year	Country/No.	TZP	CIP	LVX	SXT	TGC	MIN	RIF
Wang L/2020 [31] *	China/52	86.5%	50%	71.2%	36.5%	78.8%	100%	76.9%
Lin JN/2018 [15] *	Taiwan/92	23.9%	9.8%	52.2%	12%	23.9%	91.3%	NA
Comba IY/2022 [32] ^#^	USA/139	84%	32.1%	80.8%	90.0%	NA	100%	NA
Wu C/2024 [33] ^#^	China/71	8.5%	45.1%	64.9%	63.4%	36.6%	100%	NA

Foot notes: CIP (ciprofloxacin), LVX (levofloxacin), TZP (piperacillin–tazobactam), SXT (trimethoprim–sulfamethoxazole), TGC (tigecycline), MIN (minocycline), RIF (rifampicin), NA (not accessed), *: Broth microdilution was used to conduct antimicrobial susceptibility testing, ^#^: Agar dilution was used to conduct antimicrobial susceptibility testing.

**Table 2:** Antimicrobial susceptibility patterns of *Elizabethkingia meningoseptica*.

Author/Year	Country/No.	TZP	CIP	LVX	SXT	TGC	MIN	RIF
Hsu MS/2011 [5] ^#^	Taiwan/99	78.8%	43.4%	81.8%	91.9%	32.3%	NA	NA
Han MS/2017 [17] ^#^	Korea/17	100%	23%	35%	6%	NA	NA	94%
Lin JN/2018 [15] *	Taiwan/20	5%	10%	30%	10%	15%	60%	NA
Cheng YH/2019 [22] *	Taiwan/11	73%	0%	55%	0%	55%	100%	NA
Chang TY/2019 [34] *	Taiwan/11	NA	NA	NA	NA	NA	NA	100%
Kuo SC/2021 [20] *	Taiwan/7	14.3%	42.9%	71.4%	100%	100%	100%	100%
Comba IY/2022 [32] ^#^	USA/72	89.4%	22.9%	78.7%	89.4%	NA	100%	NA

Foot notes: CIP (ciprofloxacin), LVX (levofloxacin), TZP (piperacillin–tazobactam), SXT (trimethoprim–sulfamethoxazole), TGC (tigecycline), MIN (minocycline), RIF (rifampicin), NA (not accessed), *: Broth microdilution was used to conduct antimicrobial susceptibility testing, ^#^: Agar dilution was used to conduct antimicrobial susceptibility testing.

**Table 3:** Antimicrobial susceptibility patterns of *Elizabethkingia anophelis*.

Author/Year	Country/No.	TZP	CIP	LVX	SXT	TGC	MIN	RIF
Han MS/2017 [17] ^#^	Korea/51	92%	22%	29%	22%	NA	NA	96%
Chew KL/2018 [35] *	Singapore/79	92.4%	21.5%	78.5%	92.4%	5.1%	97.5%	NA
Lin JN/2018 [15] *	Taiwan/72	30.6%	9.7%	58.3%	12.5%	26.4%	100%	NA
Cheng YH/2019 [22] *	Taiwan/105	73%	1%	16%	4%	20%	98%	NA
Jian MJ/2019 [21] *	Taiwan/115	88%	3%	12%	8%	22%	100%	NA
Chang TY/2019 [34] *	Taiwan/142	NA	NA	NA	NA	NA	NA	94.4%
Burnard D/2020 [36] *	Australia/22	31.25%	75.0%	100%	75.0%	NA	100%	NA
Kuo SC/2021 [20] *	Taiwan/90	3.3%	5.6%	47.8%	96.7%	52.2%	100%	81.1%
Chang Y/2021 [37] *	China/39	71.8%	30.8%	38.5%	NA	10.3%	100%	20.5%
Tang HJ/2021 [38] ^#^	Taiwan/30	93.3%	26.7%	70.0%	73.3%	0%	100%	90.0%
Hu S/2022 [39] ^#^	USA/197	34.01%	48.2%	76.1%	1.02%	35.5%	100%	93.9%
Comba IY/2022 [32] ^#^	USA/18	92.9%	35.7%	71.4%	85.7%	NA	100%	NA

Foot notes: CIP (ciprofloxacin), LVX (levofloxacin), TZP (piperacillin–tazobactam), SXT (trimethoprim–sulfamethoxazole), TGC (tigecycline), MIN (minocycline), RIF (rifampicin), NA (not accessed), *: Broth microdilution was used to conduct antimicrobial susceptibility testing, ^#^: Agar dilution was used to conduct antimicrobial susceptibility testing.

**Table 4:** Antimicrobial susceptibility patterns of *Elizabethkingia miricola*.

Author/Year	Country/No.	TZP	CIP	LVX	SXT	TGC	MIN	RIF
Han MS/2017 [17] ^#^	Korea/18	94%	56%	100%	28%	NA	NA	66%
Kenna DTD/2018 [40] ^#^	UK/12	41.6%	75%	NA	100%	NA	100%	NA
Cheng YH/2019 [22] *	Taiwan/22	73%	14%	77%	18%	50%	100%	NA
Chang TY/2019 [34] *	Taiwan/14	NA	NA	NA	NA	NA	NA	85.7%
Comba IY/2022 [32] ^#^	USA/13	91.7%	50.0%	91.7%	83.3%	NA	NA	NA

Foot notes: CIP (ciprofloxacin), LVX (levofloxacin), TZP (piperacillin–tazobactam), SXT (trimethoprim–sulfamethoxazole), TGC (tigecycline), MIN (minocycline), RIF (rifampicin), NA (not accessed), *: Broth microdilution was used to conduct antimicrobial susceptibility testing, ^#^: Agar dilution was used to conduct antimicrobial susceptibility testing.

**Table 5:** Recommendation of antibiotics therapy to treat patients with infections caused by *Elizabethkingia* species.

Pathogens	TZP	CIP	LVX	SXT	TGC	MIN	RIF
*Elizabethkingia* species	?	X	?	?	X	++	?
*E. meningoseptica*	+ *	X	X	?	X	++	++
*E. anophelis*	+	X	X	?	X	++	++
*E. miricola*	?	X	++	?	X	++	?

Foot notes: CIP (ciprofloxacin), LVX (levofloxacin), TZP (piperacillin–tazobactam), SXT (trimethoprim–sulfamethoxazole), TGC (tigecycline), MIN (minocycline), RIF (rifampicin), X (not recommendation), **?** (questionable), **+** (recommendation), **++** (strong recommendation), E. (*Elizabethkingia*), * (not recommendation in Taiwan).

## Data Availability

The datasets generated during and/or analyzed during the current study are not publicly available but are available from the corresponding author on reasonable request.
